# Predicted Visual Impact of a Small Aperture Intraocular Lens in Reducing Higher Order Aberrations in Post-Radial Keratotomy Patients

**DOI:** 10.3390/vision9020046

**Published:** 2025-05-29

**Authors:** Roberta M. van den Berg, Sarah DeVaro, Karolinne Maia Rocha, Marcela Fetrin de Barros, Stephen D. Klyce

**Affiliations:** 1 Storm Eye Institute, Medical University of South Carolina, Charleston, SC 29425, USA; 2 Department of Ophthalmology, Federal University of São Paulo (UNIFESP), São Paulo 04023-062, Brazil; 3 Department of Ophthalmology, University of São Paulo (USP), São Paulo 05403-000, Brazil; 4 Department of Ophthalmology, Icahn School of Medicine at Mount Sinai, New York, NY 10029, USA; sklyce@klyce.com

**Keywords:** post-RK, higher-order aberrations, pinhole effect

## Abstract

The purpose of this study is to evaluate the potential impact of small aperture optics on corneal aberrations in post-RK patients. Preoperative data was evaluated from 32 eyes of 23 post-RK patients. Scheimpflug tomography was used to obtain measurements of corneal HOAs at 6-mm, 4-mm, and 2-mm corneal plane aperture diameters. The data was extrapolated using a non-linear fit to estimate HOAs that would be obtained with the 1.6 mm effective pinhole IOL aperture at the corneal plane for individual patients. The average RMS HOAs estimated for the 1.6 mm aperture was 0.063 ± 0.015 μm compared to 0.185 ± 0.029 μm for the natural pupil size. A postoperative RK case with an IC-8^®^ Apthera™ unilateral implantation demonstrated a 70% reduction in HOAs by objective measurement and prediction, plus a 2-line improvement in CDVA. Prediction modeling revealed that HOAs may be reduced in post-RK patients following pinhole IOL implantation, compared to the natural photopic pupil size. Furthermore, the approach can be used to guide which post-RK patients would benefit from a small aperture IOL during cataract surgery.

## 1. Introduction

Radial Keratotomy (RK) is a refractive surgical technique primarily used from the 1970s until the early 1990s to treat myopia by making a series of manual paracentral radial incisions to flatten the central cornea. The incisions made in RK were often used in conjunction with peripheral corneal arcuate incisions (AK) for astigmatic correction [1]. While it is estimated that more than a million patients underwent RK worldwide, RK has been replaced by the newer excimer and femtosecond laser surgical techniques. Although RK is no longer the preferred refractive surgical procedure, patients who previously underwent RK are now undergoing cataract surgery evaluation, and over 40% of these patients in some cohorts fail successful therapeutic interventions due to high irregular astigmatism [2].

In addition to high irregular astigmatism, challenges in this patient population include diurnal variation in refraction and visual acuity and the known late complication of progressive hyperopic shift in the oblate-shaped post-RK cornea [3]. The unique challenge to this patient population of impaired corneal stability and diurnal fluctuations in refraction leads to difficulty in maintaining the targeted post-operative refraction after cataract surgery [4]. These factors require extensive planning before cataract surgery to achieve the desired refractive target. In a post-RK cornea, intraocular lens (IOL) power predictions are complicated by the uncertainties in measuring an accurate refraction due to the increased apparent depth of focus from the higher order aberrations (HOAs) in addition to the usual errors in biometry and limitations in predicting the postoperative effective lens position (ELP) [5].

Besides making measurements more difficult, unlike other keratorefractive surgeries, radial keratotomy alters the curvature of both corneal surfaces; when present, most corneal HOAs arise from the interface between the air and corneal tear film, but with RK, both corneal surface curvatures are altered, which influences total corneal power [6].

Maximizing the size of the optical zone and optimizing its centration with any keratorefractive procedure is essential for optimal optical performance, as doing so reduces HOAs minimizing nighttime visual disturbances, such as glare and halos [7]. It is well known that larger pupillary sizes contribute to increased ocular aberrations. Even in normal healthy eyes with excellent visual acuity, a larger pupil corresponds to increased HOAs [8]. However, when a refractive procedure, trauma, or pathology impacts visual function from induced HOAs or stromal haze, an effective solution can be obtained with the pinhole effect. This effect is a well-described phenomenon where, as the pupil size decreases, the depth of focus increases and entrained ocular aberrations decrease [9,10]. This phenomenon has been applied surgically with the pupilloplasty technique that has demonstrated a significant improvement in visual acuity [11,12]. Trindade et al. published a pin hole IOL case series that included eyes with high amounts of irregular astigmatism—a history of keratoconus, RK, penetrating keratoplasty, and traumatic corneal laceration [12]. The case series followed implantation of a pinhole lens (XtraFocus, Morcher GmbH, Stuttgart, Germany) placed in the ciliary sulcus in a piggyback configuration demonstrating significantly improved vision postoperatively [12]. The pinhole effect was also used in the design of the 1.6 mm small aperture of the corneal inlay (KAMRA, Acufocus, Inc., Irvine, CA, USA) with the aim of treating presbyopia by increasing the depth of focus in one eye to improve near vision [13].

The KAMRA small aperture inlay has since been relocated for use in cataract surgery. The original corneal inlay was reconfigured with a slightly smaller 1.36 mm aperture and encapsulated in an IOL—the IC-8^®^ Apthera™ (Bausch & Lomb, Inc., Bridgewater, NJ, USA). The aperture size of 1.36 mm at the plane of the IOL was reduced to produce the same optical aperture size at the corneal plane of 1.6 mm as the corneal inlay [14]. The IOL has been approved by the FDA for unilateral implantation to extend the depth of focus in patients with up to 1.5 D of cylinder with a refractive target of −0.75 D [15,16,17].

Although the use of pinhole optics has demonstrated reduction in corneal aberrations and improvement in vision [9], the small aperture IOL has not been broadly studied in patients with highly irregular corneas, such as patients with a history of RK. This study aims to determine the impact of simulated small aperture optics on a cohort of patients post-RK. It is of interest to investigate the pinhole effect on post-RK patients undergoing cataract evaluations to determine whether a small aperture IOL should be considered for use during surgical intervention in this patient population.

## 2. Materials and Methods

A retrospective chart review was conducted to collect preoperative data from 32 eyes of all 23 post-RK patients presenting for cataract surgery seen at the Storm Eye Institute, Medical University of South Carolina (MUSC) from November 2016 to November 2022. The study was approved by the MUSC Institutional Review Board (IRB) and adhered to the tenets of the Declaration of Helsinki.

Post-RK patients were examined in the clinic for routine cataract surgery. Exclusion criteria included patients with a history of corneal disease or surgery other than RK (e.g., AK, mini-RK, keratoconus, corneal dystrophies, ocular surface disease, ocular trauma, herpetic keratitis, corneal melt, recurrent corneal erosion, corneal scarring, and nystagmus).

Refractive data, including manifest refraction spherical equivalent (MRSE), uncorrected distance visual acuity (UDVA), corrected distance visual acuity (CDVA), and uncorrected near visual acuity (UNVA) were obtained from electronic medical records. None of the patients were using scleral lenses during the examination. The Scheimpflug instrument (Pentacam^®^ HR, Oculus Optikgeräte GmbH, Wetzlar, Germany) was used to assess total corneal HOAs up to the 6th order using the device’s Zernike analysis software for apertures of 6-, 4-, and 2 mm as well as for the natural photopic pupil size. Calculations of aberrations adhered to the “Methods of Reporting Optical Aberrations of Eyes” available in ANSI Z80.28-2017. The data was extrapolated to estimate the HOAs within a 1.6 mm aperture calculated with a non-linear function for each subject [18].

The OPD-Scan III (NIDEK, Inc., Gamagori, Japan) was used in one post-RK patient not from the study cohort to simulate the impact the pinhole IOL could have with the 1.6 mm aperture on CDVA by convolving the HOAs measured from corneal topography with photopic and mesopic pupil sizes and with a 1.6 mm aperture.

For one post-RK patient who underwent cataract surgery with the implantation of the IC-8^®^ Apthera™, corneal HOAs up to the 6th order and the whole eye HOAs were measured using the Scheimpflug and with ray-tracing (iTrace, Tracey Technologies, Houston, TX, USA) instruments, respectively, both pre-operatively and 1 and 3 months post-operatively.

A *p*-value less than or equal to 0.05 was contemplated as statistically significant. Quantitative variables were constituted as mean ± SD. Pearson correlation analysis and 99% confidence intervals were acquired with Excel for MAC (v.16.8.4, Microsoft, Redmond, WA, USA).

## 3. Results

### 3.1. Main Results

Thirty-two eyes of 23 post-RK patients were identified meeting the inclusion criteria for the study. The pre-operative demographic data are presented in Table 1. Note that visual deficits were due in part to cataracts–correlations to corneal HOAs were not statistically significant consequently.

Data from the HOAs obtained from 6-, 4-, and 2-mm apertures were fit individually with non-linear regressions to estimate the HOAs that could be expected with the pinhole IOL with its 1.6 mm effective aperture at the corneal plane after implantation.

### 3.2. Figures

**Figure 1:**
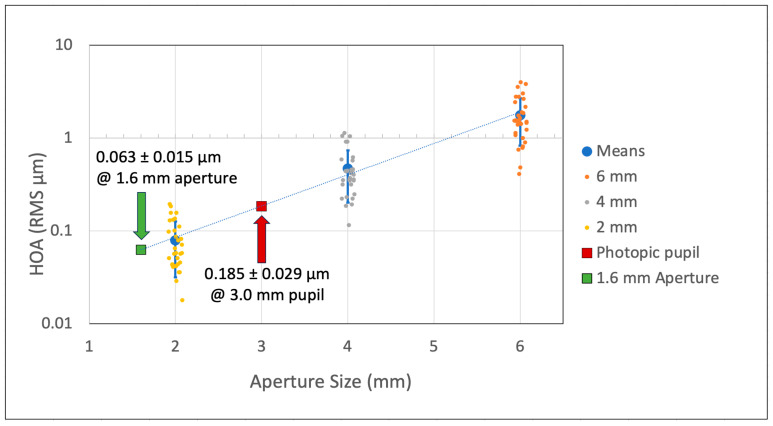
Means ± S.D. of the corneal HOAs measured for the 6-, 4-, and 2 mm apertures in post-RK cohort. Individual subject data are shown in dithered scatter plots. The mean HOAs (0.185 ± 0.029: 99% confidence interval) for the cohort at their average pupil size of 3.0 ± 0.8 mm is shown in red. At the 1.6 mm aperture, the predicted mean HOAs were 0.063 µm ± 0.015 µm (99% confidence interval).

The average RMS values for the HOAs expected for a 1.6 mm aperture for these patients was found to be 0.063 µm ± 0.015 (Figure 1).

This was a significant reduction in HOAs (*p* < 0.001) measured for the 6- and 4-mm apertures. These patients had an average photopic pupil of 3.0 ± 0.8 (range 1.7–5.1) mm. The HOAs under normal lighting (photopic) were 0.185 ± 0.029 µm, significantly greater (*p* < 0.0001) than those predicted for the IC-8 after implantation; an average reduction in HOAs of 66% was predicted for this cohort.

**Figure 2:**
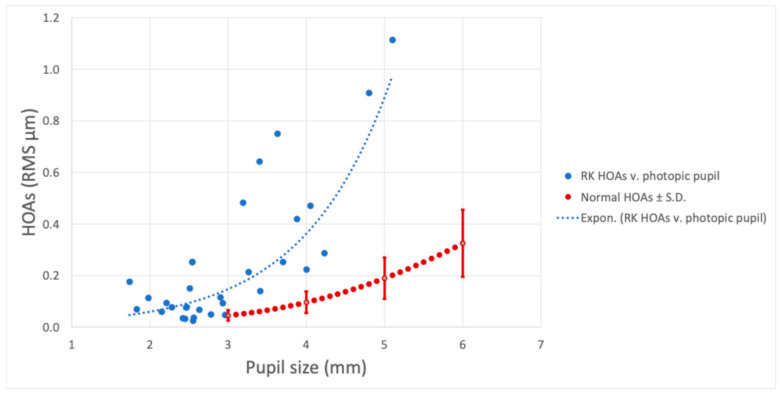
Photopic pupil aberrations in post-RK patients compared to HOAs in normal eyes. The red dotted line indicates average HOAs for normal subjects over the pupil sizes of 3–6 mm [8].

Figure 2 illustrates the total corneal HOAs measured for the natural pupil in the RK cohort, comparing these to the corneal HOAs previously measured for a large sample of normals [8]. The HOAs of some of the subjects with smaller pupils were within the range of normals; the benefit to these subjects would be an increase in depth of focus (not measured).

**Figure 3:**
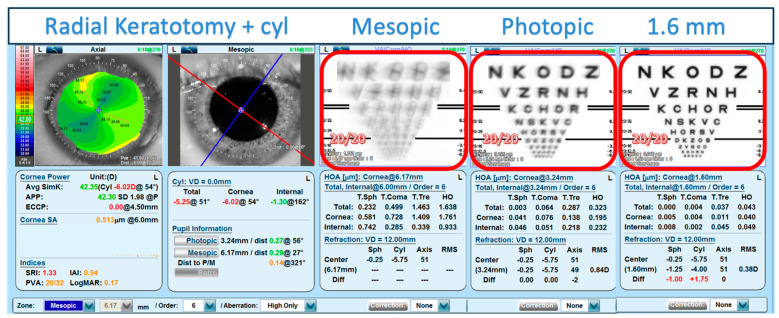
NIDEK OPD-Scan Zernike convolutions of corneal HO aberrations for different real and simulated pupil diameters (values for mesopic, photopic and 1.6 mm are shown in the red boxes). This simulation indicates the potential for CDVA 20/20 for this subject with pinhole optics. Note that the Potential Visual Acuity (PVA) derived from the Surface Regularity Index (SRI) [19] given in the left panel estimates CDVA of 20/32 for the photopic pupil is consistent with the photopic convolution.

While evaluation of corneal HOAs provides a metric to assess the optical quality of corneas after refractive surgery or other corneal conditions (e.g., keratoconus, PKP, trauma) another clinically useful tool that can be used to predict the potential improvement in vision with the pinhole effect, is to use image convolution analysis [18]. Corneal topographers and tomographers generally use the Zernike coefficient method to measure the optical wavefront. These data can then be used to convolve the measured wavefront with an image to visualize the impact of HOAs. An example of doing this with a standard ETDRS chart is shown in Figure 3 of a random post-RK subject with significant corneal aberrations. The simulated vision under mesopic and photopic pupil sizes can be demonstrated, along with the predicted vision that would be obtained with the small aperture IOL.

**Figure 4:**
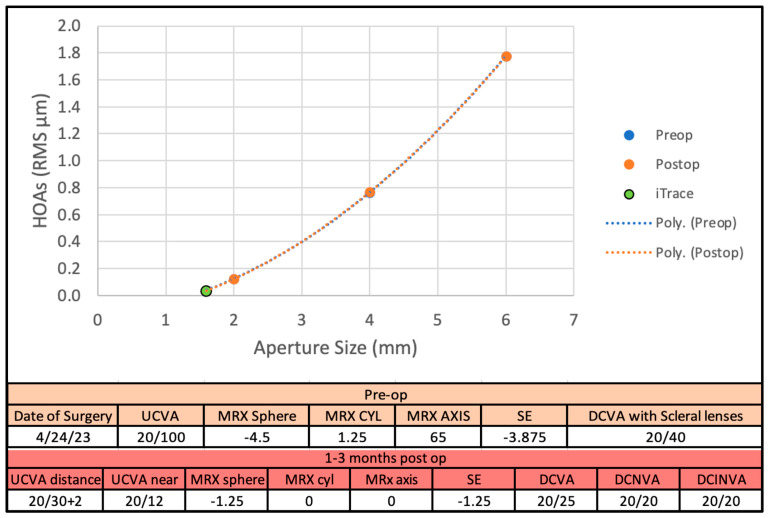
Case of a post-RK patient who underwent IC-8^®^ Apthera™ implantation during cataract surgery. Scheimpflug measurements of corneal pre- and post-operative HOAs represented by the blue and orange colors were essentially unchanged by the surgery and overlay one another. Predicted total RMS HOAs from cornea alone with a 1.6 mm aperture were: pre-op 0.040 µm, post-op 0.036 µm. Total eye HOAs measured by raytracing aberrometry (green data point) were 0.028 µm after surgery. Uncorrected and distance corrected visions improved significantly. (Case: 71 y.o.♂ OS 9 cut RK, 6 incision AK, pre/post IC-8).

Although the pinhole IOL has been used in highly irregular corneas, clinical experience after implantation is limited with the current cohort. However, the predicted improvement in vision can be illustrated in a surgical case, a post-RK 71-year-old male with a cataract in the left eye. His preoperative uncorrected visual acuity (UDVA) was 20/100 and CDVA of 20/40 (corrected with a scleral lens–showing the principal cause of vision loss to be corneal HOAs). The patient underwent cataract surgery with implantation of the IC-8^®^ Apthera™ IOL. Three months postoperatively the UCVA was 20/30^+2^, CDVA was 20/25 (without a scleral lens), and UNVA was 20/12 (Jaeger J1^+^). The predicted reduction in corneal RMS HOAs were estimated to be reduced from 0.40 μm preoperatively (for the natural photopic pupil of 3.0 mm) to ~0.040 μm estimated for the 1.6 mm aperture at the corneal plane (Figure 4).

After cataract surgery with the IC-8 implantation, total ocular RMS aberrations were measured for the whole eye through the IC-8 aperture with the ray-tracing aberrometer. The measured whole eye aberrations (with the iTrace) were 0.1 µm, within the margin of error of the predicted value and a 75% reduction of HOAs. It is of interest to note that for this patient, the cataract surgery did not induce additional corneal aberrations. As can be seen in Figure 4, pre- and post-op HOA data lie on top of one another.

## 4. Discussion

RK surgery induces a central corneal flattening as a treatment for myopia while slightly steepening the peripheral cornea. As noted above, RK performed with a variable configuration of manual corneal incisions can induce visually significant corneal HOAs in a significant proportion of subjects [5]. Thus, these eyes warrant special consideration and, like other eyes that have undergone keratorefractive procedures, specialized IOL power calculators have been developed (e.g., https://iolcalc.ascrs.org, Barret True K formulas) [20,21,22,23]. Standard keratometry values are often not accurate measures of the central corneal power particularly after refractive surgery [24]. IOL power calculators alternatively use effective refractive power or average central corneal power-type measurements available from corneal topography. Scheimpflug tomography and optical coherence tomography, which evaluate curvatures from both corneal surfaces, have also been used effectively to provide accurate measures of total corneal power especially for abnormally shaped corneas [25]. For highly aberrated corneas this is particularly important since the irregularities cannot provide accurate data without spatial averaging.

Aside from calculations, another special consideration is the pupil size of the patient. As previously discussed, an increase in pupil size is correlated with an increase in corneal aberrations measured. In patients who experience increased irregular astigmatism after RK, those with larger pupils will have a greater impact on visual quality. Our data is in accordance with a study published by Applegate et.al., which demonstrated that HOAs present in post-RK patients include visually significant fourth-order or spherical-like aberrations [26].

In our patient cohort, the average RMS value for the total corneal HOAs estimated for 1.6 mm at the corneal plane was 0.063 ± 0.015 µm compared to 0.185 ± 0.029 µm for the natural pupil size (3.0 ± 0.8 mm) in post-RK eyes. The predicted value for the 1.6 mm aperture approximates the corneal RMS HOAs measured in 1433 normal subjects by Salmon and van de Pol of 0.10 µm for a 4 mm pupil [8]. Those patients in the current study who had normally small pupils and lower amounts of HOAs (Figure 1) would likely benefit from the IC-8 not so much from the reduction in peripheral corneal aberrations provided by the 1.6 mm aperture, but from the increase in depth of focus that is a second benefit of the pinhole effect. Hence, under this condition, the post-RK patients should experience improved visual acuity with the IC-8 Apthera IOL with the expected improvement in near vision. This was confirmed in the case report presented in Figure 4 with the marked improvement in UDVA and UNVA.

It is recognized that the aberrations were measured with a Scheimpflug instrument that aligns on the corneal vertex and therefore the HOAs collected at various diameters will not be evaluated from precisely the same area of the cornea that projects through the pinhole of the IOL. Hence, if there are large variations in corneal irregular astigmatism over small areas, using the method proposed here for predict the visual benefit could be in error. This could occur. For example, if one or more of the radial RK incisions extended to near the visual axis, but this is a rare adverse event. The position of the implanted pinhole IOL has been found to be very accurately positioned and stable in measurements made at post-operative month 3 [27], and corneal irregular astigmatism tends to be relatively uniform over small distances on the corneal surface.

As noted above, benefits of a small aperture device include reduction in peripheral corneal aberrations and an increased depth of focus. One study by Ang reported implantation of the pinhole IOL in the nondominant eye yielded a CDVA of at least 20/25 with 1.5 D of astigmatic defocus [28]. Ang also demonstrated 0.25 D of extended depth of focus with bilateral implantation [29]. Charman [30] provides an overview of the impact of the pinhole effect on the depth of field comparing normal subjects to KAMRA inlay patients, as well as expanding the photopic depth of focus from ~2.75 D to ~4.5 D.

Our findings support extending the use of small aperture IOLs in highly aberrated corneas, specifically the potential benefit in the challenging post-RK cases. This is supported by previous studies. Implantation of a small aperture IOL (Xtrafocus, Morcher GmbH) has been demonstrated to be a safe and effective procedure in post-RK eyes with irregular astigmatism [31]. These findings were confirmed by Langer, who reported a case series of 17 highly aberrated corneas with good visual outcomes following cataract surgery and small-aperture IOL implantation [32].

As these lenses become utilized more frequently, it is important to discuss expectations preoperatively with patients. As demonstrated in a previous publication and in this study (Figure 3), the impact of small aperture optics on visual acuity in highly aberrated eyes can be visually displayed to patients via topographic technology [18]. Using real and simulated pupil diameters by Zernike convolutions can also aid clinicians in predicting best corrected visual acuity and improvement in HOAs with use of small aperture optics.

Despite the various advantages of the pinhole IOL, there are known limitations to small-aperture technology. Previously cited concerns include reduced visualization of the peripheral retina (retinal tears) on routine dilated fundus exams or future retinal surgical interventions as well as complications from IOL decentration within the capsular bag [33]. However, the IOL has not been found to jeopardize the visualization of the retina while improving visual function in patients with aberrated corneas [34]. A significant number of patients undergo posterior capsule opacification after cataract surgery which is treated with Nd:YAG laser capsulotomy. Because the IC-8 masks a large portion of the pupil, with carbon black nanoparticles embedded in the carrier polyvinylidene fluoride IOL, care needs to be taken to apply the laser shots within the aperture or outside its circumference [35]. One case reports that carbon nanoparticles can burst from the lens when struck by the laser leading to decreased visual acuity with the possible necessity of a lens exchange [36]. Patients may report visual disturbances, including reduced contrast sensitivity, particularly in low-light environments. These phenomena occur due to the lens design, which limits light entry and alters the way light is diffracted [37]. However, decrease in retinal illumination caused by the annular light masking in the pinhole corneal inlay can be compensated by neuroadaptation [38]. Of note, different retinal illumination between the two eyes can distort object motion (the Pulfrich effect) and may cause patients complaints when driving. However, in the Trindade case series, none of the subjects that had the IOL implanted reported this situation, as well as the reduced retinal luminance had no clinical relevance [12].

The present study has a theoretical nature, as the HOAs were measured in 6-, 4- and 2 mm pupil sizes, and calculation was made to extrapolate the values to a 1.6 mm diameter, which is the IOL aperture at the corneal plane. This methodology as well as image analysis (Figure 3) can be used clinically to help predict which cataract patients with irregular corneas would benefit from the implantation of the small aperture IOL. The additional advantage of the expected improvement in depth of focus is a secondary intended benefit for the aphakic presbyope.

## 5. Conclusions

In conclusion, convolution analysis and Zernike simulations can demonstrate the potential of small aperture technology use in patients with a history of RK, resulting in improved quality of vision. The simulation for a 1.6 mm pupil diameter showed significant reduction in measured HOAs. This reduction and, in particular, the increase in depth of focus may provide RK patients with more tolerance to diurnal fluctuations in vision due to impaired corneal stability. This technology can be a promising therapeutic option for highly aberrated corneas including not only post-RK patients, but other conditions such as keratoconus [31], post-keratoplasty, and trauma.

## Figures and Tables

**Table 1:** Radial Keratotomy patient demographics (*n* = 23).

Sex (F/M)	13/10
Age (years)	65 ± 7 (49–77) *
RK incisions	7.7 ± 2.6 (4–16) *
CDVA (LogMar)	0.21 ± 0.16 *

* Values are shown as mean ± standard deviation, in parentheses is the range.

## Data Availability

Raw data collected from patient charts is not in a segregated form, but can be made available upon request to the authors after assurance of anonymization.
